# Clinical, Laboratory, and Imaging Features of COVID-19 in a Cohort of Patients: Cross-Sectional Comparative Study

**DOI:** 10.2196/28005

**Published:** 2021-09-21

**Authors:** Rami Qaisieh, Mohammad Al-Tamimi, Naser El-Hammuri, Marwan Shalabi, Muna M Kilani, Hana Taha, Abdallah Al-Muhtaseb, Ibrahim Alfarrajin, Marwan Abu Shaqra, Almothana Hamdan

**Affiliations:** 1 Department of General and Special Surgery Faculty of Medicine Hashemite University Zarqa Jordan; 2 Department of Basic Medical Sciences Faculty of Medicine Hashemite University Zarqa Jordan; 3 Department of Pediatrics and Neonatology Faculty of Medicine Hashemite University Zarqa Jordan; 4 Global Health Development/Eastern Mediterranean Public Health Network Amman Jordan; 5 Department of Neurobiology, Care Sciences and Society Karolinska Institute Stockholm Sweden

**Keywords:** COVID-19, gender, clinical, laboratory, imaging, SARS-CoV2, Jordan

## Abstract

**Background:**

The clinical, laboratory, and imaging features of COVID-19 disease are variable. Multiple factors can affect the disease progression and outcome.

**Objective:**

This study aimed to analyze the clinical, laboratory, and imaging features of COVID-19 in Jordan.

**Methods:**

Clinical, laboratory, and imaging data were collected for 557 confirmed COVID-19 patients admitted to Prince Hamzah Hospital (PHH), Jordan. Analysis was performed using appropriate statistical tests with SPSS version 24.

**Results:**

Of the 557 COVID-19 polymerase chain reaction (PCR)-positive cases admitted to PHH, the mean age was 34.4 years (SD 18.95 years; range 5 weeks to 87 years), 86.0% (479/557) were male, 41% (29/70) were blood group A+, and 57.1% (93/163) were overweight or obese. Significant past medical history was documented in 25.9% (144/557), significant surgical history in 12.6% (70/557), current smoking in 14.9% (83/557), and pregnancy in 0.5% (3/557). The mean duration of hospitalization was 16.4 (SD 9.3; range 5 to 70) days; 52.6% (293/557) were asymptomatic, and 12.9% (72/557) had more than 5 symptoms, with generalized malaise and dry cough the most common symptoms. Only 2.5% (14/557) had a respiratory rate over 25 breaths/minute, and 1.8% (10/557) had an oxygen saturation below 85%. Laboratory investigations showed a wide range of abnormalities, with lymphocytosis and elevated C-reactive protein (CRP), erythrocyte sedimentation rate (ESR), and D-dimer the most common abnormalities. Ground glass opacity was the most common imaging finding. Men had a significantly higher frequency of symptoms, incidence of smoking, reduced hemoglobin, increased monocyte %, elevated creatinine levels, and intensive care unit admissions compared with women (*P*<.05). Hospitalization duration was associated with increased age, male gender, symptom score, history of smoking, elevated systolic blood pressure, elevated respiratory rate, and elevated monocyte %, CRP, ESR, creatinine, and D-dimer (*P*<.05).

**Conclusions:**

Most COVID-19 cases admitted to PHH were asymptomatic. Variabilities in symptoms, signs, laboratory results, and imaging findings should be noted. Increased age, male gender, smoking history, and elevated inflammatory markers were significantly associated with longer duration of hospitalization.

## Introduction

In December 2019, an outbreak of pneumonia of unknown etiology was identified in Wuhan city, China [1]. Later, it was found that the causative pathogen was severe acute respiratory syndrome coronavirus 2 (SARS-CoV2) [1]. The routes of transmission of this virus are mainly droplets and direct contact with patients, and the main source of the disease at present is patients with COVID-19 [2]. On March 11, 2020, the World Health Organization declared COVID-19 a pandemic due to its exponential spread all over the globe [3].

Studies have shown that COVID-19 is a systemic disease where different systems are affected differently; therefore, the clinical manifestations of the disease vary from patient to patient, with fever (78%-87%) and cough (57%-68%) being the most common manifestations in adults. Other manifestations like dyspnea (23%-24%), myalgia (17%-24%), and fatigue (31%-39%) are present to a lesser extent [4,5]. A small percentage of patients develop gastrointestinal symptoms such as nausea (6.0%-6.5%), vomiting (4.0%-6.5%), and diarrhea (8%-10%) [4,5]. The least prevalent symptoms are ophthalmological (2%-4%) and neurological (0%-14%) [4,5]. The severity of the disease varies among patients, with the elderly and patients with comorbidities being affected the most [6]. There are many complications of the disease such as acute respiratory distress syndrome, acute cardiac injury, acute kidney injury, and shock [7]. Patients are also at increased risk of hypercoagulability and thromboembolism [8].

X-ray imaging studies showed that bilateral involvement is more common than unilateral, and the most common lesion is a ground glass appearance followed by consolidation [4]. Computed tomography (CT) scans also confirmed these findings [9]. The most prevalent laboratory findings are decreased albumin, high C-reactive protein (CRP), lymphopenia, increased platelets, increased lactate dehydrogenase, and a high erythrocyte sedimentation rate (ESR) [10].

Although the prevalence of COVID-19 is equal between men and women, the disease is more severe in men [11]. Some studies attributed this to a higher expression of angiotensin-converting enzyme 2 (ACE2), the receptor for SARS-CoV2, in men than in women in pathological conditions [12]. Furthermore, it has been found that ACE2 expression is higher in current and ex-smokers, and smoking is more common in men than in women. Thus, the disease is more severe in men [13]. Patients with hypertension or chronic obstructive pulmonary disease (COPD) tend to have more severe COVID-19 disease. Children have less severe disease than adults, and these differences are possibly due to having different expression levels of ACE2 receptors [14]. While this disease involves mainly the respiratory tract, different organ systems can become involved.

Researchers have dug into massive gene expression datasets to show that other potential target cells that also produce ACE2 and TMPRSS2 are scattered throughout the body, which could explain the systemic nature of this disease [14]. While multiple studies have reported greater disease severity and mortality in men infected with COVID-19, no comparative studies have been conducted regarding the differences in clinical, laboratory, and imaging findings according to gender [11-13,15].

The first case of COVID-19 in Jordan was registered on March 2, 2020 in a Jordanian citizen who came back from Italy [16], and the number of cases as of December 12, 2020 exceeded 250,000 [3,16,17]. Even though there is a tremendous number of studies worldwide regarding COVID-19 patients’ clinical features, laboratory findings, and imaging findings, there are only a few in our region (the Middle East), and no study has yet been done in Jordan. Clinical, laboratory, and imaging findings are widely variable according to geographic location, disease severity, SARS-CoV2 strains, population demographics, immunity, and other factors [2,10]. The aim of this study was to describe the clinical manifestations, laboratory findings, and imaging findings of COVID-19 patients in Jordan with an emphasis on gender-related differences.

## Methods

### Study Population

A total of 557 confirmed COVID-19 cases admitted to Prince Hamzah Hospital (PHH) during the period from March 1, 2020 to August 1, 2020 were recruited prospectively to this study after giving formal voluntary consent, and they were followed daily for clinical, paraclinical, and outcome parameters. All COVID-19 cases were confirmed by at least one positive COVID-19 reverse transcription (RT)-PCR test performed by an accredited referral lab. All COVID-19 recovery cases were confirmed by complete clinical and laboratory resolution, including 2 negative COVID-19 RT-PCR tests within 2 days. The government of Jordan had a policy at the time of the study to admit all COVID-19–positive patients to the hospital for isolation regardless of symptom severity.

The study protocol was approved by the institutional review board (IRB) at the Hashemite University (No: 1∕5∕2019∕2020) and the Jordanian Ministry of Health/PHH IRB (No: 1/1631).

### Demographic, Clinical, and Laboratory Data From COVID-19 Patients in Jordan

Confirmed COVID-19 patients’ demographics; clinical, social, and medical history; and laboratory and imaging data were obtained directly from patients, relatives, or medical records of patients admitted to PHH, Amman, Jordan (the main COVID-19 isolation and management center in Jordan). Data were recorded on the first day of admission and daily during follow-ups. Demographic data included age, gender, weight, height, BMI, and blood group. Clinical data included symptoms reported by patients, vital signs, medical and surgical history, and duration of hospitalization. Laboratory data included all laboratory tests performed for patients during their admission. Imaging data assessed by an accredited radiology specialist were extracted for the 135 patients who had imaging studies (questionnaire in Multimedia Appendix 1 and primary data file in Multimedia Appendix 2 are provided as supplementary material).

### Statistical Analysis

Descriptive statistical analysis was used for determination of demographic, clinical, laboratory, and imaging findings. Percentages and means (SD) were calculated to describe the distributions of categorical and continuous variables, respectively. Chi-square and Fisher exact tests were used to assess the association between the study participants’ age, gender, BMI, and duration of hospitalization with their clinical, laboratory, and imaging data. The level of statistical significance was set at *P*≤.05. Data were analyzed using Microsoft Excel 2010 and SPSS version 24.0.

## Results

### Demographics and Clinical Features of COVID-19 in Jordan

Of the 557 COVID-19 cases who were admitted to PHH, the gender distribution was 86.0% (479/557) men and 14.0% (78/557) women. Among these patients, the mean age was 34.4 years (SD 18.95 years; range 5 weeks to 87 years), and the largest age group was 21-40 years old (190/557, 34.1%). BMI was documented in 163 patients: 8.0% (13/163) were underweight, 35.0% (57/163) were normal weight, 29.4% (48/163) were overweight, and 27.6% (45/163) were obese (Table 1). Blood groups were determined for 70 patients (70/557, 12.6%) with blood group A+ (29/70, 41%) and O+ (19/70, 27%) being the most common. Significant past medical history was documented in 25.9% (144/557) of patients, significant surgical history in 12.6% (70/557), current smoking in 14.9% (83/557), a history of allergies in 1.8% (10/557), and pregnancy in 0.5% (3/557). The mean duration of hospitalization was 16.4 (SD 9.3) days, ranging from 5 days to 70 days (Table 1).

**Table 1:** Demographic and clinical data of COVID-19 patients admitted to Prince Hamzah Hospital (PHH; n=557).

Variable		Number of participants	Relative percentage	Absolute percentage
**Age (years)**				
	<1	10	1.8	1.8
	1-20	152	27.3	27.3
	21-40	190	34.1	34.1
	41-60	154	27.6	27.6
	61-80	48	8.6	8.6
	>80	3	0.5	0.5
**Gender**				
	Male	479	86.0	86.0
	Female	78	14.0	14.0
**BMI**				
	Underweight >18.5 kg/m^2^	13	8.0	2.3
	Normal 18.5-24.9 kg/m^2^	57	35.0	10.2
	Overweight 25-29.9 kg/m^2^	48	29.4	8.6
	Obese >30 kg/m^2^	45	27.6	8.1
	ND^a^	394	N/A^b^	70.7
**Blood group**				
	A+	29	41.4	5.2
	B+	13	18.6	2.3
	AB+	2	2.9	0.4
	O+	19	27.1	3.4
	A-	1	1.4	0.2
	B-	1	1.4	0.2
	O-	5	7.1	0.9
	ND	487	N/A	87.4
**Admission duration (days)**				
	5-14	336	60.3	60.3
	15-30	177	31.8	31.8
	31-46	33	5.9	5.9
	47-70	11	2.0	2.0
**Symptoms**				
	Generalized malaise	120	21.5	21.5
	Headache	75	13.5	13.5
	Loss of smell	70	12.6	12.6
	Diarrhea	57	10.2	10.2
	Loss of taste	60	10.8	10.8
	Chills/rigors	77	13.8	13.8
	Myalgia	61	11.0	11.0
	Nasal congestion	72	12.9	12.9
	Dry cough	121	21.7	21.7
	Fever	108	19.4	19.4
	Rhinorrhea	57	10.2	10.2
	Sweating	35	6.3	6.3
	Wet cough	44	7.9	7.9
	Shortness of breath	56	10.1	10.1
	Abdominal pain	33	5.9	5.9
	Chest pain	29	5.2	5.2
	Palpitations	12	2.2	2.2
	Hemoptysis	5	0.9	0.9
	Others	48	8.6	8.6
**Symptom scores**				
	Asymptomatic	293	52.6	52.6
	Mild: 1-5	192	34.5	34.5
	Moderate: 6-10	50	9.0	9.0
	Severe: 11-17	22	3.9	3.9
**Past medical history**				
	Asthma	8	1.4	1.4
	Hypertension	25	4.5	4.5
	Diabetes	19	3.4	3.4
	Diabetes and hypertension	60	10.9	10.9
	Pregnancy	3	0.5	0.5
	Others	33	6	6
	No	408	73.9	73.2
	ND	5	N/A	0.9
**Past surgical history**				
	Yes	70	12.6	12.6
	No	487	87.4	87.4
**Allergic history**				
	Yes	10	2.6	1.8
	No	377	97.4	67.7
	ND	170	N/A	30.5
**Smoking**				
	Past smoker	12	3.6	2.2
	Current smoker	83	25.2	14.9
	Never smoked	234	71.1	42.0
	ND	228	N/A	40.9
**Heart rate (/minute)**				
	<60	2	0.4	0.4
	60-80	257	48.5	46.1
	81-128	271	51.1	48.7
	ND	27	N/A	4.8
**Systolic blood pressure (mm Hg)**				
	<120	243	47.8	43.6
	120-139	241	47.4	43.3
	140-159	21	4.1	3.8
	≥160	3	0.6	0.5
	ND	49	N/A	8.8
**Diastolic blood pressure (mm Hg)**				
	<80	374	73.6	67.1
	80-89	115	22.6	20.6
	90-99	16	3.1	2.9
	≥100	3	0.6	0.5
	ND	49	N/A	8.8
**Respiratory rate (/minutes)**				
	<12	0	0.0	0.0
	12-25	493	97.2	88.5
	>25	14	2.8	2.5
	ND	50	N/A	9.0
**Oxygen saturation (%)**				
	<80	4	0.8	0.7
	80-84	6	1.2	1.1
	85-94	52	10.0	9.3
	95-100	458	88.1	82.2
	ND	37	N/A	6.6

^a^ND: not determined.

^b^N/A: not applicable.

The patients complained of a variety of symptoms; nevertheless, most of the patients (293/557, 52.6%) were asymptomatic, while 34.5% (192/557) had 1-5 symptoms, 9.0% (50/557) had 6-10 symptoms, and 3.9% (22/557) had more than 11 symptoms. Among the symptomatic patients, generalized malaise and dry cough were the most common symptoms, and they were documented in 21.5% (120/557) and 21.7% (121/557) of the patients, respectively. These were followed by fever (108/557, 19.4%), chills and rigors (77/557, 13.8%), headache (75/557, 13.5%), nasal congestion (72/557, 12.9%), loss of smell (70/557, 12.6%), myalgia (61/557, 11.0%), loss of taste (60/557, 10.8%), rhinorrhea (57/557, 10.2%), and shortness of breath (56/557, 10.1%). Gastrointestinal symptoms were less frequently documented with diarrhea (57/557, 10.2%) and abdominal pain (33/557, 5.9%) being most prevalent. The least reported symptoms were chest pain (29/557, 5.2%), palpitations (12/557, 2.2%), and hemoptysis (5/557, 0.9%; Table 1). Regarding the vital signs of admitted COVID-19 patients, about 50% (271/557, 48.7%) of patients had a heart rate >80 beats per minute and systolic blood pressure ≥120 mm Hg (265/557, 47.6%), while nearly 25% had a diastolic pressure ≥80 mm Hg (134/557, 24.1%). Only 2.5% (14/557) had a respiratory rate over 25 per minute, with about 2% having an oxygen saturation below 85% (10/557, 1.8%; Table 1).

### Laboratory Data for COVID-19

Laboratory investigations for COVID-19 patients admitted to PHH (Table 2 and Table 3) showed low hemoglobin and hematocrit in 9.9% (55/557) and 7.7% (43/557) of patients, respectively. Total white blood cell count was low in 7.2% (40/557) and high in 4.8% (27/557) of patients. Differential count showed that the neutrophil percentage was low in 14.2% (79/557), the lymphocyte percentage was low in 12.6% (70/557) and high in 28.7% (160/557), the basophil percentage was low in 44.9% (250/557), the eosinophil percentage was low in 43.4% (242/557), and the monocyte percentage was high in 26.2% (146/557). Platelet count was low in 6.6% (37/557), with high prothrombin time, international normalized ratio, and D-dimer found in 2.3% (13/557), 3.6% (20/557), and 13.1% (73/557), respectively. Inflammatory markers including CRP and ESR were elevated in 28.7% (160/557) and 26.4% (147/557) of patients, respectively. Urea and creatinine were elevated in 3.4% (19/557) and 4.8% (27/557) respectively. Aspartate transaminase (AST), alanine transaminase (ALT), and lactate dehydrogenase (LDH) were elevated in 10.2% (57/557), 8.8% (49/557), and 5.0% (28/557), respectively. Bilirubin total and direct were elevated in 9.2% (51/557) and 4.1% (23/557), respectively. Hyponatremia and hypokalemia were found in 5.0% (28/557) and 4.1% (23/557), respectively (Table 3).

**Table 2:** Mean laboratory values from COVID-19 patients admitted to Prince Hamzah Hospital (PHH; n=557).

Investigations	Value, mean (SD)
HB^a^ (g∕dL)	13.92 (1.73)
HCT^b^ (%)	41.12 (5.18)
WBC^c^ (∕μL)	7190 (4.19)
Neutrophil (%)	52.82 (25.92)
Lymphocyte (%)	32.97 (15.99)
Basophil (%)	0.51 (0.35)
Monocyte (%)	9.17 (4.10)
Eosinophil (%)	1.59 (1.82)
Platelets (count∕μL)	251,900 (107,320)
CRP^d^ (mg/L)	14 (38)
ESR^e^ (mm/h)	28.27 (28.96)
PT^f^ (s)	13.54 (2.80)
INR^g^	1.05 (0.18)
Urea (mmol/L)	4.74 (2.13)
Creatinine (mmol/L)	74.08 (70.52)
Sodium (mmol/L)	125.77 (40.04)
Potassium (mmol/L)	4.68 (8.29)
AST^h^ (U/L)	27.64 (23.15)
ALT^i^ (U/L)	24.21 (22.18)
LDH^j^ (U/L)	248.62 (301.74)
ALP^k^ (U/L)	89.74 (66.82)
D-dimer (µg/mL)	0.524 (0.865)
Ferritin (ng/mL)	161.32 (262.51)
Bilirubin (total; µmol/L)	11.52 (5.49)
Bilirubin (direct; µmol/L)	2.73 (4.81)

^a^HB: hemoglobin.

^b^HCT: hematocrit.

^c^WBC: white blood cell.

^d^CRP: C-reactive protein.

^e^ESR: erythrocyte sedimentation rate.

^f^PT: prothrombin time.

^g^INR: international normalized ratio.

^h^AST: aspartate transaminase.

^i^ALT: alanine transaminase.

^j^LDH: lactate dehydrogenase.

^k^ALP: alkaline phosphatase.

**Table 3:** Laboratory data from COVID-19 patients admitted to Prince Hamzah Hospital (PHH; n=557).

Investigations			Number of patients	Relative percentage	Absolute percentage
**HB^a^ (g∕dL) categories**					
	Low <12	55		10.3	9.9
	Normal 12-16	418		78.3	75.0
	High >16	61		11.4	11.0
	ND^b^	23		N/A^c^	4.1
**HCT^d^ (%) categories**					
	Low <35	43		8.1	7.7
	Normal 35-47	435		81.5	78.1
	High >47	56		10.5	10.1
	ND	23		N/A	4.1
**WBC^e^ (∕μL) categories**					
	Low <4000	40		7.5	7.2
	Normal 4000-11,000	466		87.4	83.7
	High >11,000	27		5.1	4.8
	ND	24		N/A	4.3
**Neutrophil (%) categories**					
	Low <40	79		14.8	14.2
	Normal 40-80	430		80.5	77.2
	High >80	25		4.7	4.5
	ND	23		N/A	4.1
**Lymphocyte (%) categories**					
	Low <20	70		13.1	12.6
	Normal 20-40	304		56.9	54.6
	High >40	160		30.0	28.7
	ND	23		N/A	4.1
**Basophil (%) categories**					
	Low <0.5	250		46.6	44.9
	Normal 0.5-1	258		48.1	46.3
	High >1	28		5.2	5.0
	ND	21		N/A	3.8
**Monocyte (%) categories**					
	Low <2	4		0.8	0.7
	Normal 2-10	383		71.9	68.8
	High >10	146		27.4	26.2
	ND	24		N/A	4.3
**Eosinophil (%) categories**					
	Low <1	242		45.3	43.4
	Normal 1-6	276		51.7	49.6
	High >6	17		3.0	3.1
	ND	22		N/A	3.9
**Platelets (count∕μL) categories**					
	Low <150,000	37		6.9	6.6
	Normal 150,000-450,000	488		91.6	87.6
	High >450,000	8		1.5	1.4
	ND	24		N/A	4.3
**CRP^f^ (mg/L) categories**					
	Normal 0-5.0	322		66.3	57.8
	High >5.0	160		33.2	28.7
	ND	75		N/A	13.5
**ESR^g^ (mm/h) categories**					
	Normal 0-15	142		49.1	25.5
	High >20	147		50.9	26.4
	ND	268		N/A	48.1
**PT^h^ (s) categories**					
	Low <12	16		5.5	2.9
	Normal 12-16	262		90.0	47.0
	High >16	13		4.5	2.3
	ND	266		N/A	47.8
**INR^i^ categories**					
	Low <0.85	1		0.4	0.2
	Normal 0.85-1.15	255		92.4	45.8
	High >1.15	20		7.2	3.6
	ND	281		N/A	50.4
**Urea (mmol/L) categories**					
	Low <2.86	39		7.8	7.0
	Normal 2.86-8.2	444		88.4	79.7
	High >8.2	19		3.8	3.4
	ND	55		N/A	9.9
**Creatinine (mmol/L) categories**					
	Low <59	161		31.1	28.9
	Normal 59-104	329		63.3	59.1
	High >104	27		5.2	4.8
	ND	40		N/A	7.2
**Sodium (mmol/L) categories**					
	Low <135	28		5.5	5.0
	Normal 135-152	479		94.5	86.0
	High >152	0		0.0	0.0
	ND	50		N/A	9.0
**Potassium (mmol/L) categories**					
	Low <3.5	23		4.5	4.1
	Normal 3.5-5.3	476		93.9	85.5
	High >5.3	8		1.6	1.4
	ND	50		N/A	9.0
**AST^j^ (U/L) categories**					
	Normal ≤38	414		87.9	74.3
	High >38	57		12.1	10.2
	ND	86		N/A	15.4
**ALT^k^ (U/L) categories**					
	Normal ≤41	443		90.0	79.6
	High >41	49		10.0	8.8
	ND	65		N/A	11.7
**LDH^l^ (U/L) categories**					
	Low <125	13		3.6	2.3
	Normal 125-378	321		88.7	57.6
	High >378	28		7.7	5.0
	ND	195		N/A	35.0
**ALP^m^ (U/L) categories**					
	Low <40	15		5.7	2.7
	Normal 40-150	226		85.3	40.6
	High >150	24		9.1	4.3
	ND	292		N/A	52.4
**D-dimer (µg/mL) categories**					
	Normal <0.5	265		78.4	47.6
	High >0.5	73		21.6	13.1
	ND	219		N/A	39.3
**Ferritin (ng/mL) categories**					
	Low <12	11		3.7	2.0
	Normal 12-300	255		86.7	45.8
	High >300	28		9.5	5.0
	ND	263		N/A	47.2
**Bilirubin (total; µmol/L) categories**					
	Low <3	9		2.7	1.6
	Normal 3-16	269		81.8	48.3
	High >16	51		15.5	9.2
	ND	228		N/A	40.9
**Bilirubin (direct; µmol/L) categories**					
	Normal <5.1	282		92.5	50.6
	High >5.1	23		7.5	4.1
	ND	252		N/A	45.2

^a^HB: hemoglobin.

^b^ND: not determined.

^c^N/A: not applicable.

^d^HCT: hematocrit.

^e^WBC: white blood cell.

^f^CRP: C-reactive protein.

^g^ESR: erythrocyte sedimentation rate.

^h^PT: prothrombin time.

^i^INR: international normalized ratio.

^j^AST: aspartate transaminase.

^k^ALT: alanine transaminase.

^l^LDH: lactate dehydrogenase.

^m^ALP: alkaline phosphatase.

### Radiological Features of COVID-19

The following radiological data were obtained for 135 COVID-19 patients. CT scan studies of the chest showed that the most common appearance of infiltrates was ground glass opacity (44/135, 32.6%), followed by broncho-alveolar consolidation (14/135, 10.4%). Central involvement was noticed in 7.4% (10/135) of the patients, while peripheral involvement was observed in 26.0% (35/135) of the patients. Also, 25.2% (34/135) of the patients had lesions that were located posteriorly, in comparison to 8.1% (11/135) who had anterior lesions and 25.2% (34/135) who had mediastinal lymphadenopathy. The most affected lobe was the right lower lobe (38/135, 28.1%), followed by the left lower lobe (33/135, 24.4%), left upper lobe (23/135, 17.0%), right upper lobe (22/135, 16.3%), and right middle lobe (20/135, 14.8%; Table 4).

**Table 4:** Clinical imaging data from a chest computed tomography scan for COVID-19 patients admitted to Prince Hamzah Hospital (PHH; n=135).

Variable		Number of patients	Relative percentage	Absolute percentage
**Patterns of infiltrates**				
	Ground glass opacity	44	32.6	7.8
	Broncho-alveolar consolidation	14	10.4	2.5
	Crazy paving	4	3.0	0.7
	Subpleural retraction	3	2.2	0.5
	Bronchiectasis	2	1.5	0.4
	Vascular dilatation	0	0.0	0.0
**Central vs peripheral**				
	Central	10	7.4	1.8
	Peripheral	35	26.0	6.3
**Anterior vs posterior**				
	Anterior	11	8.1	2.0
	Posterior	34	25.2	6.1
Pleural effusion		0	0.0	0.0
Mediastinal lymphadenopathy		34	25.2	6.1
**Affected lobe**				
	Right upper lobe	22	16.3	4.0
	Right middle lobe	20	14.8	3.6
	Right lower lobe	38	28.1	6.8
	Left upper lobe	23	17.0	4.1
	Left lower lobe	33	24.4	5.9

X-ray scans showed that 3 patients (3/135, 2.2%) had solitary infiltrates, while 20 patients (20/135, 14.8%) had multiple infiltrates. Also, it showed that 4 patients (4/135, 3.0%) had peripheral lesions, 3 patients (3/135, 2.2%) had central lesions, and 1 patient (1/135, 0.7%) had both peripheral and central lesions. Regarding the most affected lung lobes, the data showed the following: left lower lobe (17/135, 12.6%), right lower lobe (21/135, 15.6%), right middle lobe (17/135, 12.6%), right upper lobe (12/135, 8.9%), and left upper lobe (12/135, 8.9%). Only 1 patient (1/135, 0.7%) had affected lung apices, and only 2 patients (2/135, 1.5%) had a pleural effusion. No patient had hilar involvement or a widened mediastinum (Table 5).

**Table 5:** Clinical imaging data from a chest x-ray for COVID-19 patients admitted to Prince Hamzah Hospital (PHH; n=135).

Variable		Number of patients	Relative percentage	Absolute percentage
Hilum affected		0	0.0	0.0
**Infiltration**				
	Solitary	3	2.2	0.5
	Multiple	20	14.8	3.5
	ND^a^	112	83.0	20.1
**Central vs peripheral**				
	Central	3	2.2	0.5
	Peripheral	4	3.0	0.7
	Both	1	0.7	0.2
	ND	127	94.1	22.8
**Affected lung lobes**				
	Right upper lobe	12	8.9	2.2
	Right middle lobe	17	12.6	3.1
	Right lower lobe	21	15.6	3.8
	Left upper lobe	12	8.9	2.2
	Left middle lobe	16	11.9	2.9
	Left lower lobe	17	12.6	3.1
Affected lung apices		1	0.7	0.2
Pleural effusion		2	1.5	0.4
Widened mediastinum		0	0.0	0.0

^a^ND: not determined.

### Associations Between Age, Gender, BMI, Hospitalization Duration and COVID-19 Clinical, Laboratory, Imaging Data

Men had a significantly higher frequency of having symptoms (symptom score) than women (244/479, 51.0% vs 19/78, 24.4%, *P*=.004). Furthermore, generalized malaise, diarrhea, chills/rigors, dry cough, rhinorrhea, and fever were significantly more frequent in men than in women (*P*≤.05). Mean heart rate and frequency of elevated heart rate were significantly higher in men than in women (*P*=.02). Past medical, past surgical, allergy, and smoking history were significantly higher in men than in women (*P*≤.001). Hemoglobin, hematocrit, monocyte %, basophile %, and creatinine levels were significantly higher in men than in women (*P*<.05), while ESR, alkaline phosphatase (ALP), and D-dimer levels were significantly higher in women than in men (*P*≤.05). Hospitalization duration and intensive care unit (ICU) admissions were significantly higher in men than in women (*P*=.000); 7 men and 1 woman were admitted to the ICU, and 2 men died. Table 6 shows the associations between age, gender, BMI, and hospitalization duration in relation to symptoms and signs, laboratory data, and imaging findings.

**Table 6:** Associations between age, gender, BMI, hospitalization duration and COVID-19 clinical, laboratory, imaging data.

Variable			*P* value for the associations					
			Age	Gender		BMI		Hospitalization duration
**Symptoms**								
	Dry cough	.03		.02	.84		.000	
	Fever	.07		.03	.06		.000	
	Wet cough	.27		.13	.53		.001	
	Chills/rigors	.99		.003	.31		.000	
	Sweating	.99		.27	.61		.004	
	Generalized malaise	.01		.000	.23		.000	
	Myalgia	.40		.054	.19		.000	
	Shortness of breath	.001		.46	.23		.000	
	Headache	.02		.08	.22		.000	
	Hemoptysis	.72		.64	.23		.25	
	Diarrhea	.013		.04	.17		.000	
	Chest pain	.001		.27	.97		.002	
	Abdominal pain	.10		.34	.57		.003	
	Palpitations	.001		.53	.66		.01	
	Loss of taste	.000		.26	.48		.000	
	Loss of smell	.000		.23	.77		.006	
	Rhinorrhea	.003		.03	.02		.000	
	Nasal congestion	.14		.20	.16		.000	
	Symptom severity score	.03		.004	.047		.000	
**History**								
	Past medical history	.000		.001	.50		.000	
	Past surgical history	.000		.001	.42		.001	
	Allergy	.24		.000	.41		.000	
	Smoking	.000		.000	.19		.000	
**Signs**								
	Heart rate	.13		.02	.60		.000	
	Systolic blood pressure	.000		.42	.02		.001	
	Diastolic blood pressure	.008		.28	.04		.000	
	Respiratory rate	.08		.13	.87		.001	
	O_2_ saturation	.000		.89	.74		.000	
**Laboratory**								
	Hemoglobin	.000		.000	.57		.34	
	Hematocrit	.000		.000	.72		.61	
	WBC^a^	.07		.31	.70		.000	
	Neutrophils	.000		.25	.26		.12	
	Basophils	.32		.046	.50		.72	
	Monocytes	.31		.02	.62		.000	
	Eosinophils	.000		.33	.61		.002	
	Lymphocytes	.000		.70	.23		.09	
	Platelets	.002		.47	.62		.35	
	CRP^b^	.000		.51	.000		.006	
	ESR^c^	.000		.000	.03		.04	
	PT^d^	.04		.86	.97		.46	
	INR^e^	.35		.91	.30		.000	
	Urea	.000		.08	.16		.39	
	Creatinine	.000		.000	.000		.01	
	Sodium	.000		.77	.41		.79	
	Potassium	.04		.49	.30		.25	
	AST^f^	.005		.61	.41		.05	
	ALT^g^	.02		.14	.02		.06	
	LDH^h^	.045		.91	.11		.12	
	ALP^i^	.000		.04	.000		.07	
	D-dimer	.000		.01	.10		.24	
	Ferritin	.000		.26	.44		.51	
	Total bilirubin	.70		.31	.11		.67	
	Direct bilirubin	.33		.06	.93		.005	
**Imaging**								
	Chest x-ray	.000		.52	.02		.25	
	CT^j^ scan conclusion	.001		.89	.003		.72	
**Other**								
	Hospitalization duration	.000		.001	.91		N/A^k^	
	Age	N/A		.23	.000		.000	
	Patient gender	.23		N/A	.16		.001	
	BMI	.000		.16	N/A		.91	

^a^WBC: white blood cells.

^b^CRP: C-reactive protein.

^c^ESR: erythrocyte sedimentation rate.

^d^PT: prothrombin time.

^e^INR: international normalized ratio.

^f^AST: aspartate transaminase.

^g^ALT: alanine transaminase.

^h^LDH: lactate dehydrogenase.

^i^ALP: alkaline phosphatase.

^j^CT: computed tomography.

^k^N/A: not applicable.

Increased age was significantly associated with a higher frequency of symptoms (symptom score; *P*=.03); increased frequency of generalized malaise, headache, loss of smell, diarrhea, loss of taste, rhinorrhea, wet and dry cough, shortness of breath, chest pain, and palpitations; higher frequency of significant past medical, past surgical, and smoking history; and increased blood pressure, lower oxygen saturation, and higher BMI (*P*≤.05). Furthermore, increased age was significantly associated with elevated CRP, ESR, urea, creatinine, ALT, and ALP levels and positive imaging findings (*P*≤.05; Table 6). Higher BMI was associated with increased age; higher symptom score; elevated blood pressure, CRP, ESR, creatinine, ALT, and ALP levels; and positive imaging findings (*P*≤.05; Table 6). Hospitalization duration was positively associated with increased age, male gender, higher symptom score, history of smoking, significant past medical and surgical histories, elevated systolic blood pressure, elevated respiratory rate, lower oxygen saturation, elevated monocyte %, elevated CRP and ESR, increased creatinine, and elevated D-dimer (*P*<0.05; Table 6).

## Discussion

### Principal Findings

Jordan has successfully managed to contain the first wave of the SARS-CoV2 virus by implementing early lockdowns. The lockdown began on March 18, 2020, when the number of known cases of the virus was less than 20. Jordan closed its borders on March 16, 2020 and kept arriving passengers in quarantine. Extensive contact tracing was carried out, and every person who tested positive for the virus was admitted to the hospital to control the spread of the virus [17]. These measures resulted in Jordan having fewer COVID-19 cases per capita compared with other countries in the region and around the world. By May 15, 2020, Jordan had 58 cases per 1 million population (1 M pop) and 0.9 deaths/1 M pop, compared with Portugal, which has about the same population and had 2776 cases/1 M pop and 116/1 M pop death, and with Greece, with 266 cases/1 M pop and 15 deaths/1 M pop. Neighboring Saudi Arabia had 1349 cases/1 M pop and 8 deaths/1 M pop [3,16-18].

Having all COVID-19–positive patients admitted to the hospital for isolation provided an opportunity to study the clinical and laboratory characteristics in patients with SARS-CoV2 viral infection in Jordan. PHH in Jordan was the main hospital designated to admit patients positive for the SARS-CoV2 virus. The patients were admitted regardless of their symptoms. In Jordan, most cases were in the age range of 21-40 years (34.1%), which is comparable to other studies [2,19]. Furthermore, a meta-analysis later in the pandemic by Pormohammad et al [4] had a mean age of 48 years for patients from studies around the world.

In this study, the younger age group (0-20 years old) represented about 29% of the cases, which was higher than the percentiles of young people infected in Saudi Arabia and China, where the percentages were about 15% [2,19]. More recently, in the United States, children under 18 years old represented 12% of all COVID-19 cases [20]. In South Korea, where all patients with positive tests were also admitted, only 9% of the patients were under 20 years of age, with the population under 24 years old representing about 24% of the nation’s population [21]. The age group under 20 years old represents about 44% of the Jordanian population [18], and this is the most likely reason for this higher percentage of COVID-19 cases among the young. Also likely contributing to this is the fact that all patients with positive tests were admitted, and extensive contact tracing was carried out.

There were more men than women in this study (86% men), which is different from other studies that either showed a slightly increased percentage of male patients [4,19] or, in a more recent meta-analysis of 3 million patients, showed equal infection rates between the 2 sexes [22]. This difference is difficult to explain but could be caused by the fact that SARS-CoV2 infection was mainly contracted by travelers and men working in the trucking business who then spread the disease to their family members [16]. Regarding the symptoms of COVID-19 in this study, most patients were asymptomatic (52.6%), and among symptomatic patients, dry cough (21.7%) and generalized malaise (21.5%), followed by fever (19.4%), were the most prevalent symptoms. This is quite similar to other studies, including 2 meta-analyses that showed that fever and cough were the most common symptoms [10,23]. Less common symptoms such as headache (13.5%), rhinorrhea (10.2%), and diarrhea (10.2%) were reported at much higher percentages in this study [24]. This is most likely explained by the fact that all patients with SARS-CoV2 viral infection were admitted regardless of symptoms, whereas other studies mainly included patients hospitalized due to their symptoms.

In this study, only 13.1% of COVID-19 patients had lymphopenia, while 30% had lymphocytosis and 56.9% of patients had a normal lymphocyte count. This contrasts with most other studies that tended to show an association between lymphopenia and COVID-19 [4,10]. Some studies hypothesized that lymphopenia may correlate with disease severity, such that lymphocyte count could possibly be used as a prognostic factor for COVID-19 patients [25,26]. Since more than half of the patients in our study were asymptomatic, this may explain the low percentage of COVID-19 patients found to have lymphopenia.

Inflammatory markers in COVID-19 patients in our study, including CRP, ESR, and LDH, were inconsistent with the findings of 2 meta-analyses [4,10]. This is most likely due to the high percentage of asymptomatic (52.6%) patients in this study. This finding increases the possibility of a positive association between high inflammatory markers and the severity of COVID-19, as proposed by yet another meta-analysis [27]. Abnormal liver enzymes, including AST and ALT, were present at lower rates compared with the results found elsewhere [28].

The radiological data from CT and x-ray scans of 135 patients were collected and analyzed. The most common lesion detected by CT scan was ground glass appearance, and this is consistent with what was found in the meta-analysis done by Bao et al [9], but at a much lower rate than those authors found (32.6% vs 90.35%, respectively). Peripheral involvement was more common than central involvement, and posterior involvement was more common than anterior involvement. These findings are similar to what was found by another study [29]. Multilobar distribution was more common than unilobar distribution, and the lower lobes were more affected than the upper lobes. Other studies found similar results [30,31]. The majority of patients who underwent chest x-ray had normal results, while Wong et al [31] found that 69% of the patients had abnormal findings on their chest radiography. This may be related to the fact that the majority of the patients in our study were asymptomatic.

When comparing male patients to female patients admitted with SARS-CoV2 infection, it was noted that male patients were more symptomatic than female patients (51.0% vs 24.4%, *P*<.05). Men were also more likely to be admitted to ICUs. In a meta-analysis that compared around 3 million patients from around the world [22], men had higher rates of ICU admission and mortality as well. The reason for this difference in morbidity and mortality between the sexes may be due to differences in the adaptive and innate immune systems, as the adaptive immune system in women has a higher number of CD 4 T cells [32,33] and stronger CD 8 cytotoxic activity [34]. Women also have more B cells and antibody production [32,35]. The reason for these differences is due to X-linked genes that affect the immune response to viruses [15,35].

Age was associated with increased symptoms (*P*<.05) and abnormal lab results. This has been documented in many other studies [36,37]. Age is also related to increased comorbidities, and in 1 meta-analysis in which there was an attempt to control for comorbidities, age itself remained a weak risk factor [38]. In our study, having an increased BMI was associated with having more symptoms, and this finding is similar to other studies and meta-analyses [39,40].

This study is the first to address the clinical, laboratory, and radiological features of COVID-19 patients in Jordan, and it was conducted with 557 patients, a considerable number of participants. A downside of this study is that all of the participants were from 1 center (PHH). Also, the data regarding laboratory testing and imaging were incompletely collected.

### Conclusions

This is the first study to describe in detail all the clinical, laboratory, and imaging findings of the first 557 confirmed COVID-19 patients admitted to PHH in Jordan. Most cases were asymptomatic, male, and overweight or obese. Generalized malaise and dry cough were the most common symptoms. Only 2.5% had a respiratory rate over 25 breaths/minute, and 2% had an oxygen saturation below 85%. Lymphocytosis and elevated CRP, ESR, and D-dimer were the most common laboratory abnormalities, while ground glass opacity was the most common imaging finding. Men had a significantly higher frequency of symptoms, smoking, abnormal laboratory findings, and ICU admissions compared to women. Hospitalization duration was positively correlated with increased age, male gender, symptom score, history of smoking, elevated systolic blood pressure, elevated respiratory rate, elevated monocyte %, and elevated CRP, ESR, creatinine, and D-dimer.

## Appendix

Multimedia Appendix 1 Data collection sheet.

Multimedia Appendix 2 Demographic, clinical, laboratory, and imaging data of COVID-19 patients.

## Abbreviations

ACE2: angiotensin-converting enzyme 2
ALP: alkaline phosphatase
ALT: alanine transaminase
AST: aspartate transaminase
COPD: chronic obstructive pulmonary disease
CRP: C-reactive protein
CT: computed tomography
ESR: erythrocyte sedimentation rate
ICU: intensive care unit
IRB: institutional review board
LDH: lactate dehydrogenase
PCR: polymerase chain reaction
PHH: Prince Hamzah Hospital
RT-PCR: reverse transcription polymerase chain reaction
